# Editorial Comment on “Risk Model for Lymph Node‐Positive Prostate Cancer After Radical Prostatectomy”

**DOI:** 10.1111/iju.70166

**Published:** 2025-07-01

**Authors:** Yozo Mitsui, Koichi Nakajima

**Affiliations:** ^1^ Department of Urology Toho University Faculty of Medicine Tokyo Japan

The significance of pelvic lymph node dissection (PLND) in regard to therapeutic outcome of patients undergoing a radical prostatectomy (RP) procedure for intermediate‐ or high‐risk prostate cancer (PC), including the necessity of an extended PLND procedure, remains unclear [1]. Furthermore, a positive lymph node metastasis (pN1) diagnosis following an RP is associated with postoperative disease progression and poor prognosis. Therefore, there is no doubt that PLND is desirable when emphasis is placed on diagnostic significance. For patients with pN1 PC after an RP, immediate androgen deprivation therapy (ADT), deferred ADT, or either of those along with radiotherapy is often planned, as previous studies have shown that such additional treatment generally results in a better outcome [2, 3]. On the other hand, further research is needed to establish proper eligibility criteria for these adjuvant therapies as well as optimal treatment strategies for patients with pN1 PC.

Tashiro et al. [4] conducted a multicenter study in Japan involving more than 500 pN1 PC patients and successfully constructed a nomogram to predict 60‐month survival following an RP procedure. Their results identified four factors associated with postoperative metastasis‐free survival (MFS); high grade, high pathological stage (pT3b or higher), large number of positive lymph nodes, and lymph node metastasis (LNM) diameter > 2 mm. Each has been shown to be related to poor prognosis in previous studies, and their use in combination was found to make more accurate prognosis predictions for individual pN1 PC patients possible. However, as the authors noted, the study has several limitations, including its retrospective nature, lack of details regarding adjuvant treatment, and use of MFS instead of overall survival as the primary endpoint. Nevertheless, the authors of the present editorial comments consider that this study provides valuable information regarding the first nomogram developed to accurately determine the prognosis of pN1 PC patients after RP. In addition, the findings presented are important for clinicians when selecting patients with pN1 PC as candidates for earlier and/or more intensive adjuvant therapy.

Regarding the significance of PLND for PC, one of the most discussed patient characteristics related to its suitability is small LNM diameter. In the study conducted by Tashiro et al. [4], 27.5% of patients with an LNM diameter < 2 mm showed a good prognosis, similar to the 26% reported by Falkenbach et al. [5]. Additionally, the study by Falkenbach et al. noted that small size as well as low number of LNMs were inversely correlated with adverse clinical parameters, with those patients showing better prognosis than others diagnosed as pN1. In other words, it is assumed that in such cases, cancer cells are completely eliminated by removal of LNMs and no additional treatment is required. Unfortunately, LNMs of this size are often difficult to detect, even with PSMA‐PET/CT imaging [5]. Therefore, PLND during RP may provide the greatest benefit for pN1 PC cases affected by LNMs with a small diameter, which are difficult to diagnose based on preoperative imaging results.

## Author Contributions

Yozo Mitsui: article writing and manuscript reviewing/editing. Koichi Nakajima: manuscript reviewing.

## Conflicts of Interest

Koichi Nakajima is a member of the Editorial Board of *International Journal of Urology* and a coauthor of this article. To minimize bias, Dr. Nakajima was excluded from all editorial decision‐making related to acceptance of the present article for publication.
